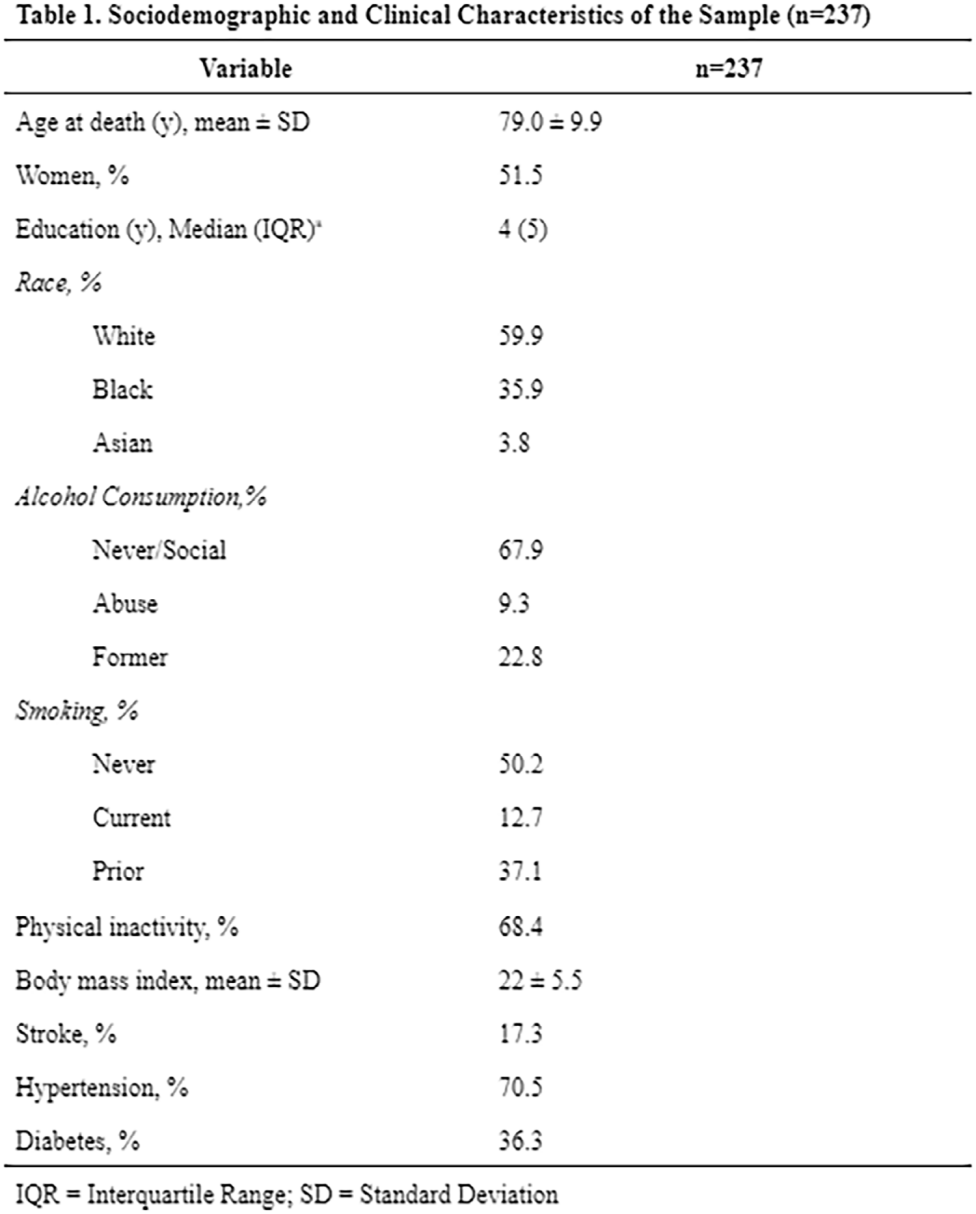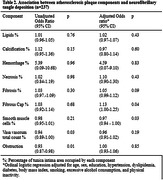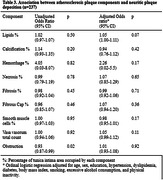# Correlation Between Atherosclerotic Carotid Plaque Composition and the Neuropathological Lesions of Alzheimer’s Disease: Partial Results

**DOI:** 10.1002/alz.085964

**Published:** 2025-01-03

**Authors:** Maria Eduarda Barbosa, Renata Elaine Paraizo Leite, Lea T. Grinberg, Ricardo Nitrini, Carlos Augusto Pasqualucci, Wilson Jacob‐Filho, Daniela Souza Farias‐Itao, Beatriz Carvalho Pontes, Maria Eduarda Braga, Alberto Fernando Oliveira Justo, Claudia Kimie Suemoto

**Affiliations:** ^1^ University of São Paulo Medical School, São Paulo, Selecione Brazil; ^2^ Biobank for aging studies of the University of São Paulo, São Paulo Brazil; ^3^ University of São Paulo Medical School, São Paulo Brazil; ^4^ Weill Institute for Neurosciences, University of California San Francisco, San Francisco, CA USA; ^5^ Cognitive and Behavioral Neurology Unit ‐ University of São Paulo, São Paulo Brazil; ^6^ Faculdade de Medicina da Universidade de São Paulo, São Paulo Brazil; ^7^ University of Sao Paulo, Sao Paulo Brazil; ^8^ Biobank for Aging Studies of the University of São Paulo, São Paulo Brazil; ^9^ University of São Paulo Medical School, São Paulo, São Paulo Brazil; ^10^ Division of Geriatrics, Department of Internal Medicine, University of Sao Paulo Medical School, São Paulo, São Paulo Brazil

## Abstract

**Background:**

Previous studies suggest an association between Alzheimer’s disease and carotid artery atherosclerosis. However, the association between atherosclerotic carotid plaque composition and Alzheimer’s disease pathology (neuritic plaques and neurofibrillary tangles) has not been explored yet.

**Method:**

Carotid arteries were dissected and the segments with the largest obstruction in the carotid bifurcation, and the common and internal carotid arteries were obtained. Each segment was immersed in paraffin and stained using Hematoxylin‐Eosin (HE) and Masson’s Trichrome. Each histological slide was photographed using a stereomicroscope (SMZ 1000; Nikon). We analyzed artery obstruction, fibrous cap, the number of vasa vasorum, and plaque components, using the software Atherosclerotic Plaque Analyzer. We characterized the neuritic plaque deposition in the brain using the Consortium to Establish a Registry for Alzheimer’s Disease (CERAD) criteria and neurofibrillary tangle deposition using the Braak & Braak criteria. We performed ordinal logistic regression models adjusted for age, sex, education, hypertension, diabetes, dyslipidemia, smoking, excessive alcohol consumption, body mass index, and physical inactivity.

**Result:**

Cross‐sectional data from 237 subjects from the Biobank for Aging Studies of the University of Sao Paulo Medical School were analyzed (mean age = 79.0±9.9 yo, 51.5% women, 59.9% white, 35.9% black and 3.8% Asian). As the percentage of smooth muscle cell area increased, neurofibrillary tangles deposition decreased (OR = 0.97, 95% CI = 0.94‐1.00, p = 0.03). Similarly, as the percentage of fibrous cap area increased, neurofibrillary tangles deposition increased (OR = 1.13, 95% CI = 1.00‐1.25, p = 0.04).

**Conclusion:**

Given that the fibrous cap is typically present in advanced atherosclerosis, and considering that smooth muscle cell area was solely assessed in the absence of well‐established atherosclerotic plaque, our examination of atherosclerotic plaque composition in carotid artery segments suggests a connection between atherosclerosis development and the accumulation of neurofibrillary tangles in the brain. We found an association with the fibrous cap proportion of the tunica intima area and an inverse association with the proportion of smooth muscle cells in the tunica intima area.